# Impact of gut microbiota on nonalcoholic fatty liver disease: insights from a leave-one-out cross-validation study

**DOI:** 10.3389/fmicb.2023.1320279

**Published:** 2024-01-08

**Authors:** Tongtong Pan, Lihuang Su, Yiying Zhang, Fangfang Yi, Yongping Chen

**Affiliations:** ^1^Hepatology Diagnosis and Treatment Center, The First Affiliated Hospital of Wenzhou Medical University and Zhejiang Provincial Key Laboratory for Accurate Diagnosis and Treatment of Chronic Liver Diseases, Wenzhou, China; ^2^The First Affiliated Hospital of Wenzhou Medical University, Wenzhou, China; ^3^Alberta Institute, Wenzhou Medical University, Wenzhou, China

**Keywords:** gut microbiota, nonalcoholic fatty liver disease, Mendelian randomization, colonystimulating factor 2 receptor β, fucosyltransferase 2, 17-beta-hydroxysteroid dehydrogenase 14

## Abstract

**Introduction:**

Enteric dysbacteriosis is strongly associated with nonalcoholic fatty liver disease (NAFLD). However, the underlying causal relationship remains unknown. Thus, the present study aimed to investigate the relationship between gut microbiota and NAFLD using Mendelian randomization (MR) and analyze the target genes potentially regulated by specific microbiota.

**Methods:**

Bidirectional two-sample MR analysis was performed using inverse variance weighted (IVW) supplemented by MR-Egger, weighted median, simple mode, and weighted mode methods. Data were pooled from gut microbiota and NAFLD association studies. The least absolute shrinkage, selection operator regression, and the Support Vector Machine algorithm were used to identify genes regulated by these intestinal flora in NAFLD. The liver expression of these genes was verified in methionine choline-deficient (MCD) diet-fed mice.

**Results:**

IVW results confirmed a causal relationship between eight specific gut microbes and NAFLD. Notably, the order Actinomycetales, NB1n, the family Actinomycetaceae, Oxalobacteraceae and the genus *Ruminococcaceae UCG005* were positively correlated, whereas Lactobacillaceae, the *Christensenellaceae R7 group*, and *Intestinibacter* were negatively correlated with NAFLD onset. In NAFLD, these eight bacteria regulated four genes: colony-stimulating factor 2 receptor β, fucosyltransferase 2, 17-beta-hydroxysteroid dehydrogenase 14, and microtubule affinity regulatory kinase 3 (*MAPK3*). All genes, except *MARK3*, were differentially expressed in the liver tissues of MCD diet-fed mice.

**Discussion:**

The abundance of eight gut microbiota species and NAFLD progression displayed a causal relationship based on the expression of the four target genes. Our findings contributed to the advancement of intestinal microecology-based diagnostic technologies and targeted therapies for NAFLD.

## 1 Introduction

Nonalcoholic fatty liver disease (NAFLD) is a metabolic syndrome characterized by less than 5% lipid deposits in hepatocytes, without alcohol consumption or other factors that may induce liver damage. The progressive stages of NAFLD include fatty liver (steatosis), nonalcoholic steatohepatitis (NASH), and fibrosis/cirrhosis, which eventually progress to end-stage liver failure or hepatocellular carcinoma with a low survival rate and a poor prognosis. Unfortunately, the global prevalence of NAFLD has considerably increased from 25.24% in 2016 to 32.4% in 2022 (Younossi et al., 2016; Riazi et al., 2022).

Advances in high-throughput sequencing technology have revealed strong associations between changes in the intestinal microbiome and the development of NAFLD. Several factors, including bidirectional crosstalk via the anatomical and physiological structure of the intestine–liver axis, disruption of the intestinal mucosal barrier, gut microbiota dysbiosis, harmful bacteria, and metabolite translocation, can promote the development of NAFLD lesions (Albillos et al., 2020). Currently, there are no specific drugs are approved for the treatment of NAFLD. However, interventions targeting intestinal microecology have exhibited therapeutic effects by rectifying intestinal dysbacteriosis, repairing damaged mucosal barriers, and regulating microbial metabolite production to alleviate chronic inflammation, insulin resistance, and oxidative stress (Craven et al., 2020; Juárez-Fernández et al., 2021; Zhao et al., 2021; Pan et al., 2022). However, the established associations between intestinal microbiota and NAFLD are based on cross-sectional studies that are prone to confounding factors and reverse causality. Thus, other study designs must be used to establish the causality between the intestinal microbiota and NAFLD and identify therapeutic targets.

Randomized controlled trials are the gold standard for investigating causality in epidemiology; however, their implementation is difficult owing to ethical constraints, high costs, and time constraints (Hariton and Locascio, 2018). Mendel’s law of inheritance dictates that parental alleles are randomly assigned to offspring, achieving the necessary randomization akin to randomized controlled trials without being affected by common confounding factors, including acquired or prior exposure, while excluding reverse causality. Mendelian randomization (MR) leverages genetic variation as an instrumental variable (IV), presenting an effective method for studying causal relationships between risk factors and outcomes (Hemani et al., 2018). Several genome-wide association studies (GWASs) on the intestinal microbiota and NAFLD (Bonder et al., 2016; Wang B. et al., 2016; Wang J. et al., 2016; Anstee et al., 2020; Ghodsian et al., 2021; Kurilshikov et al., 2021) have highlighted the relationships between these entities.

In the present study, we investigated the potential causality between different intestinal microbiota changes and NAFLD via a two-sample MR analysis using summary statistics from a GWAS. The least absolute shrinkage and selection operator (LASSO) regression and the Support Vector Machine (SVM) algorithm were used to screen the positively regulated genes in the intestinal flora. The expression of these genes was verified in the intestinal and liver tissues of mice fed a methionine choline-deficient (MCD) diet. This study aimed to outline a framework for developing gut microbiome-based diagnostic, preventive, and therapeutic approaches for NAFLD based on the target bacteria or genes they regulate.

## 2 Materials and methods

### 2.1 Data sources

Controls (770,180) and NAFLD cases (8,434) from a GWAS-based meta-analysis provided the summary statistics for NAFLD (Kurilshikov et al., 2021). Summary data for the 16S fecal microbiomes were obtained from a microbiome-based GWAS with 18,340 cases (Ghodsian et al., 2021). The GSE24807 Series Matrix File was downloaded from the Gene Expression Omnibus database.^1^ The data file (annotated as GPL2895) included the expression profile data of 17 patients, including 5 in the control group and 12 in the NAFLD group. The ethics review committees authorized each study cited in the GWAS and informed consent was obtained from all participants.

### 2.2 Study design

MR analysis was performed to determine the causal relationship between the intestinal microbes and NAFLD. Quality control experiments, such as heterogeneity and gene multiplicity tests, were conducted to confirm the reliability of causality results. Three hypotheses were assumed in the MR analysis: IVs are closely related to exposure, confounding variables that affect ‘exposure–outcome’ are not linked to IVs, and IVs affect outcome only through exposure.

### 2.3 Selection of IVs

Single-nucleotide polymorphisms (SNPs) with genome-wide importance in the gut microbiota were selected for pooling. The following selection criteria were used to choose the IVs: (1) In the forward MR analysis, *p* < 1.0 × 10^−5^ of SNPs was selected as the potential IVs; and in the reverse MR analysis, *p* < 5.0 × 10^−6^ of SNPs was selected as the potential IVs (Burgess et al., 2017). (2) The linkage disequilibrium parameter (r2) was set at a threshold of 0.001, and a genetic distance of 10,000 kb was required to select SNPs (Cheng et al., 2020). (3) The strength of the selected SNPs was assessed after removing palindromic SNPs. The strength of genetic variation as IVs was assessed using the F-statistic, and an F-statistic ≥10 indicates a strong IV with a low likelihood of introducing significant bias in the analysis (Shang et al., 2013).

### 2.4 MR analysis and sensitivity analysis

**Figure 1:**
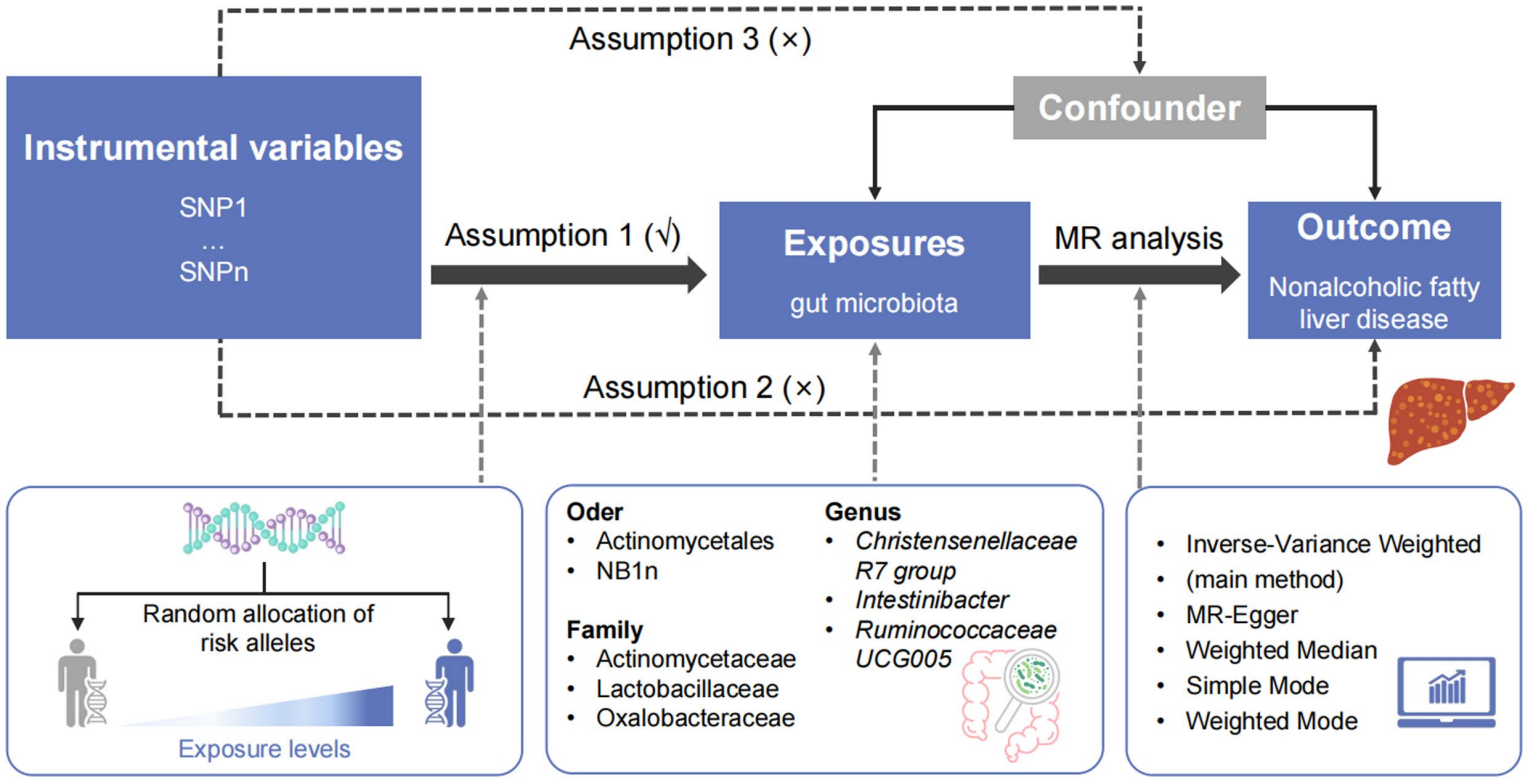
Overview of the study design. SNP, single-nucleotide polymorphism; MR, Mendelian randomization.

Chain imbalance analysis, MR analysis, and quality control experiments were performed using the R statistical programming language and two-sample MR (Hemani et al., 2018). Five methods were used to estimate causal effects (Bowden et al., 2015; Verbanck et al., 2018; Parker et al., 2020): inverse variance weighted, MR-Egger, weighted median, simple mode, and weighted mode. IVW served as the main method. The leave-one-out approach was used for sensitivity analysis and to compute the values of the remaining cumulative effects of the SNPs (Curtis et al., 2019). Horizontal genetic pleiotropy tests were performed using the intercept terms from the MR-Egger regression to determine whether IVs affected the outcomes through pathways other than exposure. The relationship between the gut microbiome and the risk of developing NAFLD was summarized as odds ratios (ORs) and 95% confidence intervals (95% CIs). A threshold of statistical significance was set at a Bonferroni-corrected *p* < 0.05 to address the issue of multiple comparisons. Figure 1 presents an overview of the analysis pipeline.

### 2.5 Feature selection process using LASSO regression and the SVM algorithm

The IVs listed in Supplementary Table S1 were converted to the corresponding SNPs using the “gwasrapidd” software package. LASSO logistic regression and the SVM algorithm were used to select the diagnostic markers for diseases using the “glmnet” and “e1071” software packages. After extracting SNPs, glmnet function of “glmnet” software package was used to fit linear model, family was set to binomial, alpha was set to 1, and then cv.glmnet was used for 10 cross-validation. When λ value reached the minimum, gene results were output.

### 2.6 Animals and diets

Twelve seven-week-old male wild-type C57BL/6 mice were purchased from the SLAC Laboratory Animal Co., Ltd. (Shanghai, China). All mice were housed under specific pathogen-free laboratory animal barrier environmental conditions with a 12/12 h light/dark cycle, temperature of 24 ± 2°C, and humidity of 50 ± 10%. Mice were randomly analyzed in two groups with six mice in each group. The control group was fed normal chow (NC) and had *ad libitum* access to water. Mouse models of NASH were established by feeding the mice an MCD diet (A02082002B; Research Diets, New Brunswick, NJ, United States) for 4 weeks. *n* = 6 per group. All experiments were conducted in accordance with the established guidelines and were approved by the Institutional Animal Care and Use Committee of Wenzhou Medical University (No. wydw2018-0252).

### 2.7 Quantitative real-time polymerase chain reaction

Total RNA was isolated from the liver samples and cultured cells using TRIzol™ reagent (Invitrogen). RNA was reverse-transcribed into cDNA using the High-Capacity cDNA Reverse Transcription Kit (Takara, Shiga, Japan). qRT-PCR was performed using SYBR Green Master Mix (Thermo Fisher Scientific) in the Real-Time PCR System (Thermo Fisher Scientific) using cDNA. β-actin was used as the invariant control. The RT-qPCR results were analyzed using the comparative Ct method (2^−∆∆Ct^). The primer sequences used for PCR are listed in Supplementary Table S2.

### 2.8 Histopathology

The liver tissues were fixed with 4% paraformaldehyde. All histological analyses were performed using paraffin-embedded tissue sections. The sections were stained with hematoxylin and eosin (Sigma, MO, United States) according to a standard procedure to visualize the lipid accumulation pattern. The Histological Activity Index (HAI) scores were quantified using ImageJ software (version 1.8.0, National Institutes of Health, Bethesda, MD, United States).

### 2.9 Biochemical analyses

Serum was isolated from blood via centrifugation at 1,300 rpm for 10 min and stored at −80°C until use. The levels of serum triglycerides (TG), total cholesterol (TC), non-esterified fatty acid (NEFA), aspartate transaminase (AST), and alanine transaminase (ALT) were measured according to the manufacturer’s instructions (Nanjing Jiancheng Bioengineering Institute, Jiangsu, China).

### 2.10 Statistical analyses

The experimental data were analyzed using GraphPad Prism 10.1.0.316 for Windows (San Diego, CA, United States). The Shapiro–Wilk test showed that the data were normally distributed, so the data were presented as the mean ± standard deviation. Then, Student’s *t*-test and one-or two-way analysis of variance were used to compare the data between groups. Statistical significance was set at *p* < 0.05.

## 3 Results

### 3.1 Selection of genetic IVs

SNPs were selected using GWAS summary statistics (Ghodsian et al., 2021). Ninety-eight SNPs were used as IVs for eight gut microbes based on the selection criteria for IVs. Supplementary Table S1 lists the details of the selected IVs.

### 3.2 Causal effects of gut microbiota on NAFLD

**Figure 2:**
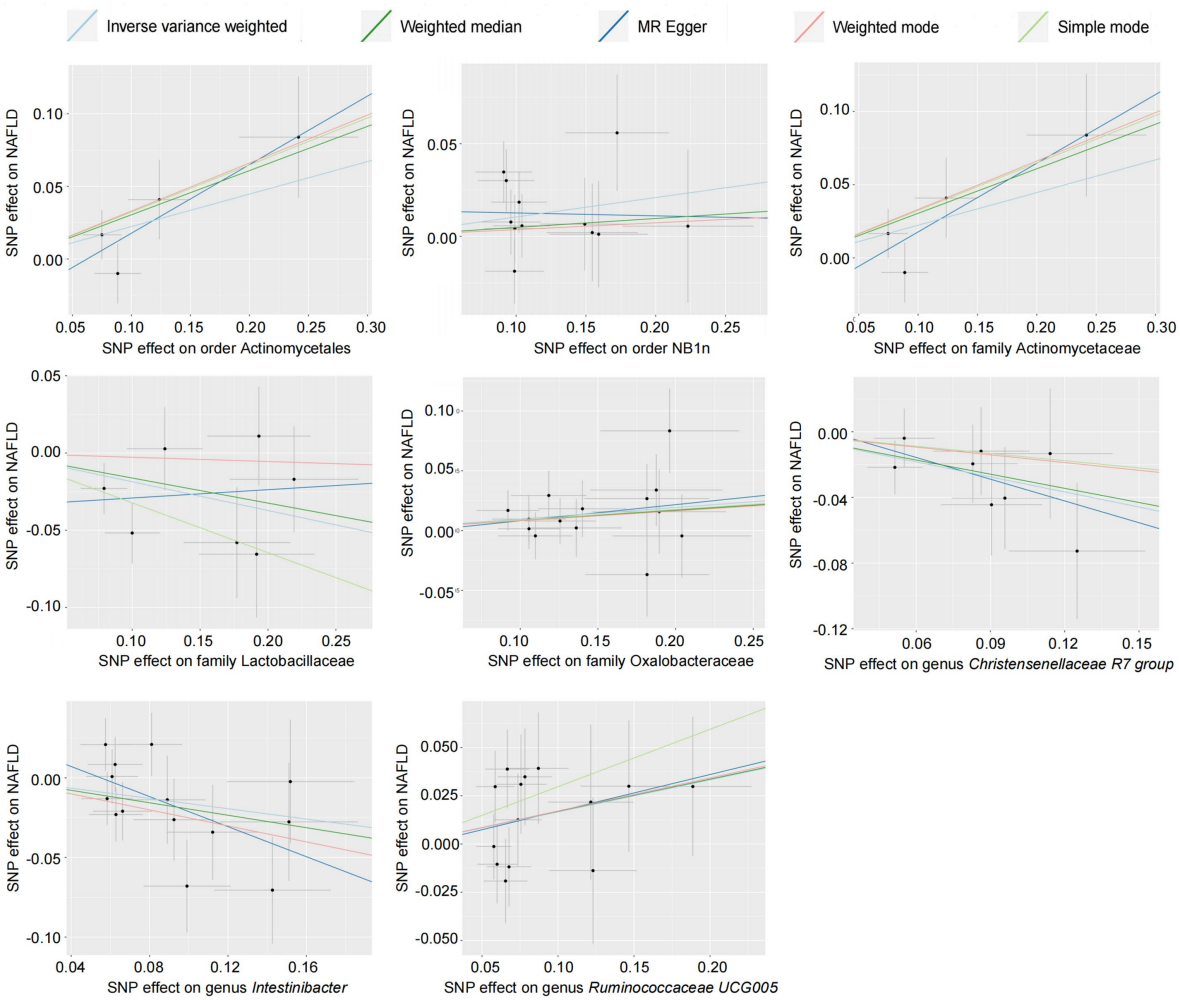
Scatter plot of the gut microbiota and NAFLD (forward results). SNP, single-nucleotide polymorphism; NAFLD, nonalcoholic fatty liver disease.

The causal relationship between the relative abundance of enterobacteria and NAFLD was analyzed at three levels (family, genus, and order) using 52-sample MR methods, primarily based on the IVW method. A total of eight intestinal microorganisms were found to be associated with NAFLD (Figure 2).

**Figure 3:**
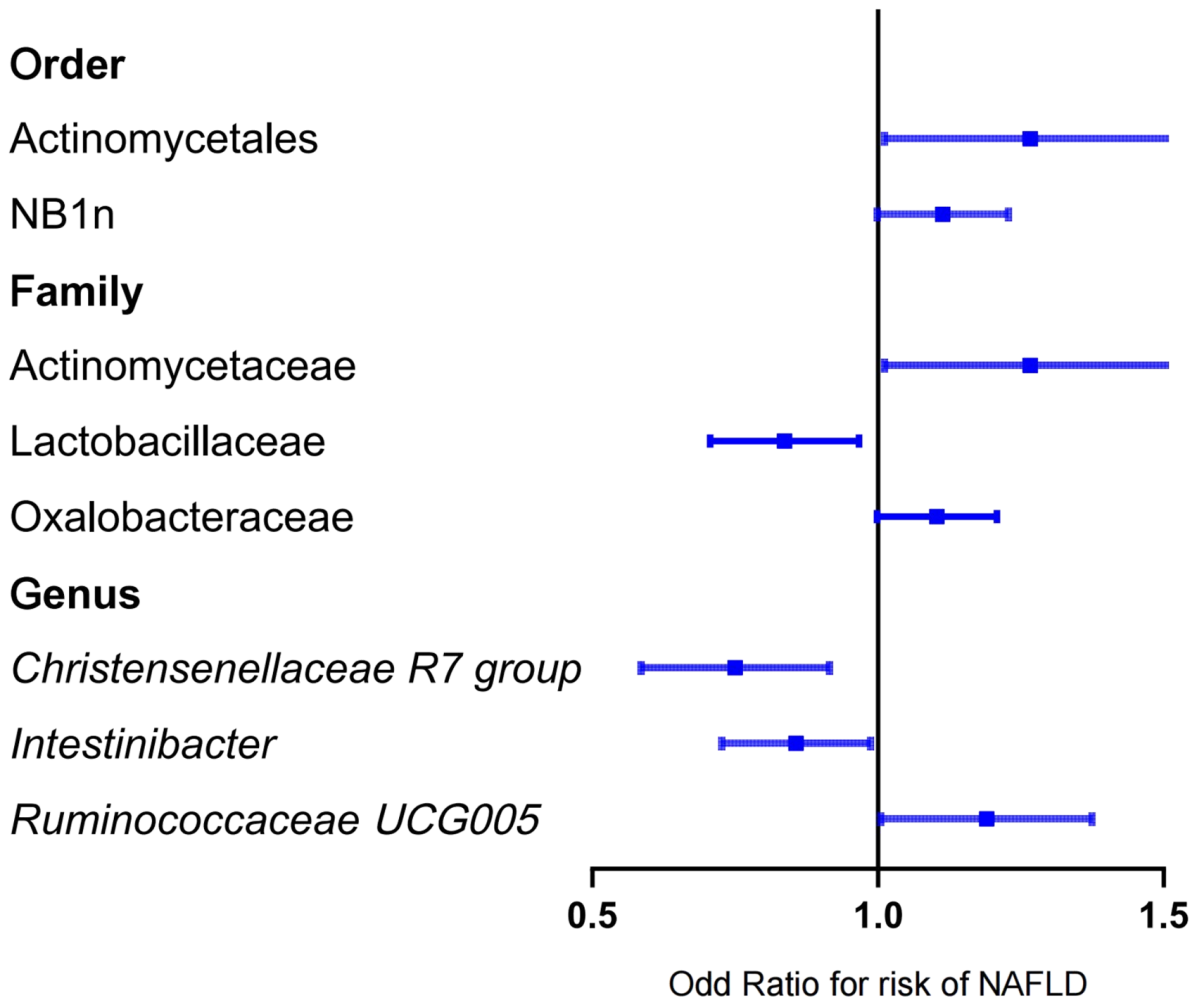
Mendelian randomization results of the positive association between gut microbiota and NAFLD based on the inverse variance weighted method. NAFLD, nonalcoholic fatty liver disease; OR, odds ratio.

**Figure 4:**
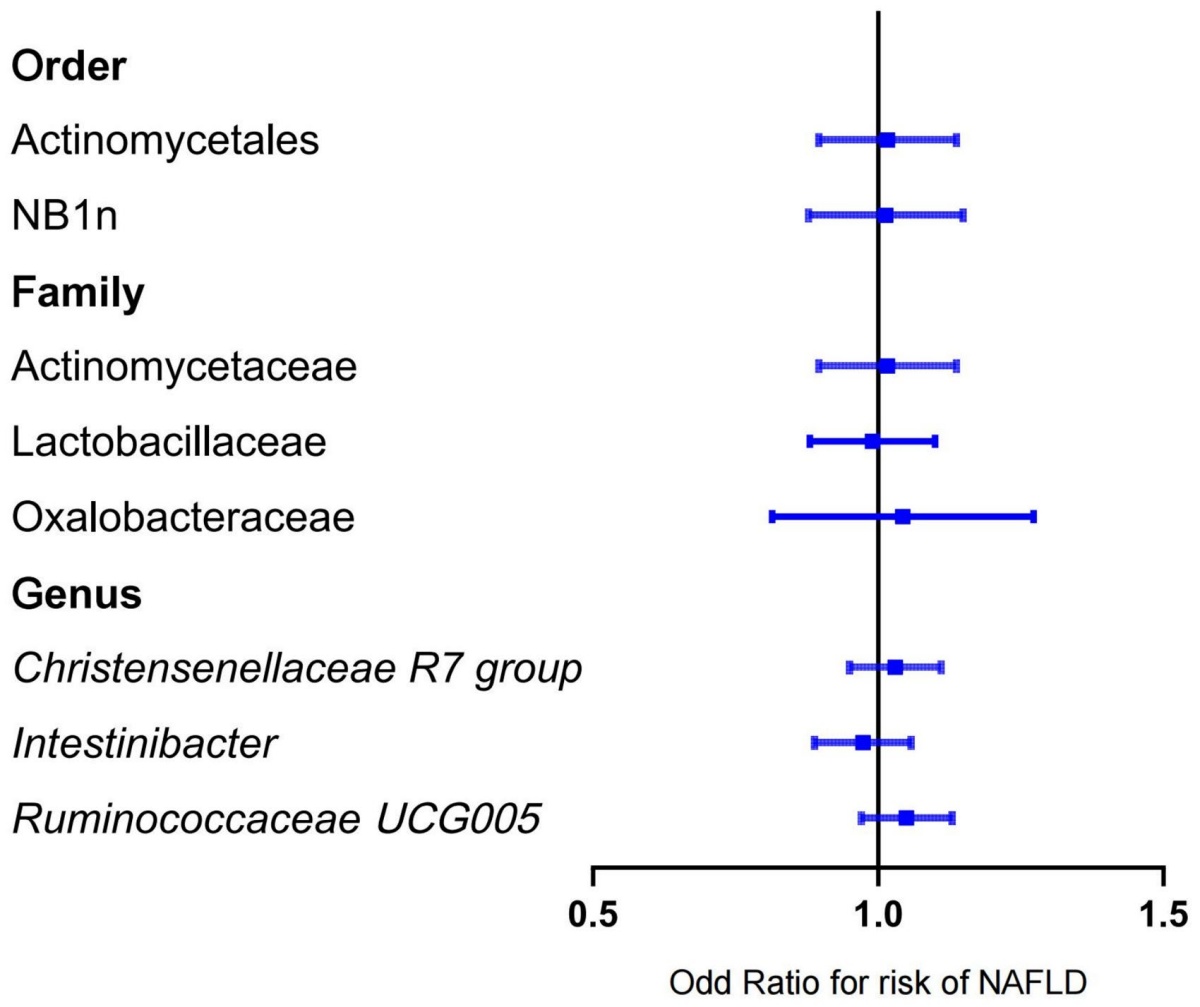
Mendelian randomization results of the negative association between gut microbiota and NAFLD based on inverse variance weighted method. NAFLD, nonalcoholic fatty liver disease; OR, odds ratio.

Forward MR analysis of the gut microbiota and NAFLD revealed that Actinomycetaceae, Oxalobacteraceae, *Ruminococcaceae UCG005*, Actinomycetales, and NB1n were positively associated with NAFLD. In contrast, Lactobacillaceae, *Christensenellaceae R7 group*, and *Intestinibacter* were negatively associated with NAFLD (Table 1, Figure 3, and Supplementary Table S3). Reverse MR analysis of the NAFLD-to-gut-microbiota ratio was performed to further evaluate the confounding factors and verify the relationship between enterobacteria and NAFLD. We observed that NAFLD had no effect on the eight gut microbiota mentioned above (Table 2, Figure 4, and Supplementary Table S4).

**Table 1:** Forward MR analysis of gut microbiota and NAFLD risk based on the IVW method.

Gut microbiota	SNP (*n*)	OR	95% CI	*p* value
Order				
Actinomycetales	4	1.25	1.02–1.53	0.03
NB1n	12	1.11	1.00–1.23	0.04
Family				
Actinomycetaceae	4	1.25	1.02–1.53	0.03
Lactobacillaceae	7	0.83	0.71–0.97	0.02
Oxalobacteraceae	14	1.10	1.01–1.21	0.04
Genus				
*Christensenellaceae R7 group*	8	0.74	0.59–0.92	0.01
*Intestinibacter*	14	0.85	0.73–0.99	0.03
*Ruminococcaceae*	14	1.18	1.01–1.38	0.03

CI: confidence interval; IVW, Inverse variance weighted; MR, Mendelian randomization; NAFLD, nonalcoholic fatty liver disease; OR: odds ratio; SNP: single-nucleotide polymorphisms.

**Table 2:** Reverse MR results for the gut microbiota causally associated with NAFLD based on the IVW method.

Gut microbiota	SNP (*n*)	OR	95% CI	*p-*value
Order				
Actinomycetales	3	1.01	0.90–1.14	0.89
NB1n	5	1.01	0.88–1.15	0.91
Family				
Actinomycetaceae	3	1.01	0.90–1.14	0.87
Lactobacillaceae	6	0.99	0.88–1.10	0.79
Oxalobacteraceae	2	0.86	0.97–1.30	0.97
Genus				
*Christensenellaceae R7 group*	5	1.03	0.95–1.11	0.47
*Intestinibacter*	4	0.97	0.89–1.06	0.53
*Ruminococcaceae*	3	1.05	0.97–1.13	0.25

CI: confidence interval; IVW, Inverse variance weighted; MR, Mendelian randomization; NAFLD, nonalcoholic fatty liver disease; OR: odds ratio; SNP: single-nucleotide polymorphisms.

### 3.3 Sensitivity analysis of the reliability of the conclusion

**Figure 5:**
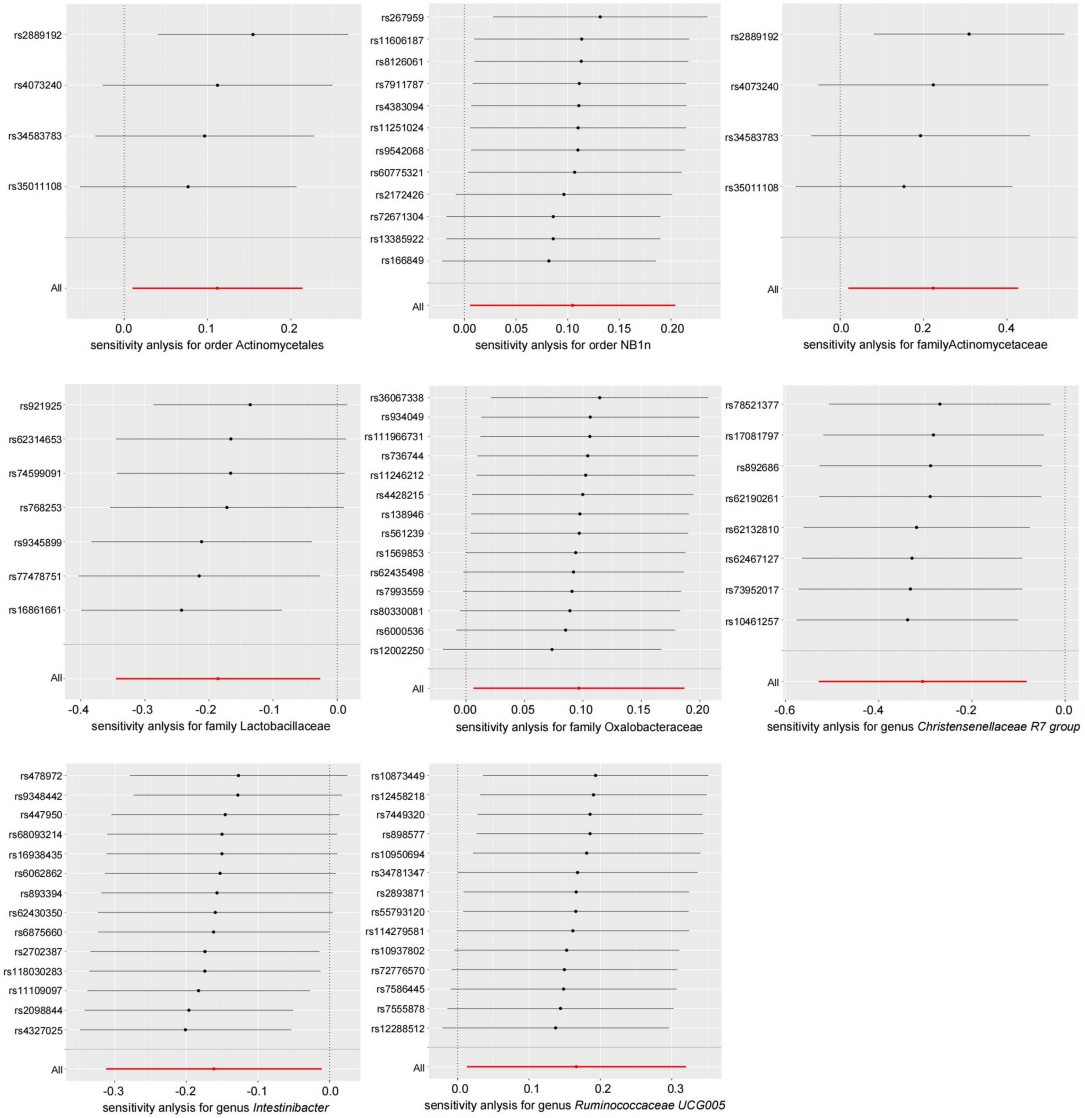
Leave-one-out plot of gut microbiota and NAFLD (forward results). NAFLD, nonalcoholic fatty liver disease.

According to the results of the MR analysis (Figure 2) and leave-one-out analysis (Figure 5) of all clusters based on different SNPs for Actinomycetaceae, Actinomycetales, *Christensenellaceae R7 group*, *Intestinibacter*, and *Ruminococcaceae UCG005*, all MR analyses were consistent with the primary analysis and the MR-Egger regression intercept term test. The Egger regression intercept test indicated that bacterial pleiotropy did not introduce bias to the results (*p* > 0.05). The results of the primary and reliability analyses using leave-one-out cross-validation supported these findings (Figure 5). Oxalobacteraceae exhibited consistent results in the multiple MR tests (Figure 2), and reliability analysis from the leave-one-out method (Figure 5) confirmed the plausibility of the outcomes. The MR-Egger regression intercept term test indicated that multipotency did not affect the abundance of Lactobacillaceae. The findings for NB1n were consistent with the main results except for those of the MR-Egger test, indicating that multipotency did not introduce bias to the results (*p >* 0.05). As expected, the results of the reliability analysis using the leave-one-out method were consistent with those of the primary analysis (Figure 5).

### 3.4 Identification of the key SNP-harboring genes regulated by positive gut microbiota and verification of their expression in mice with NASH

**Figure 6:**
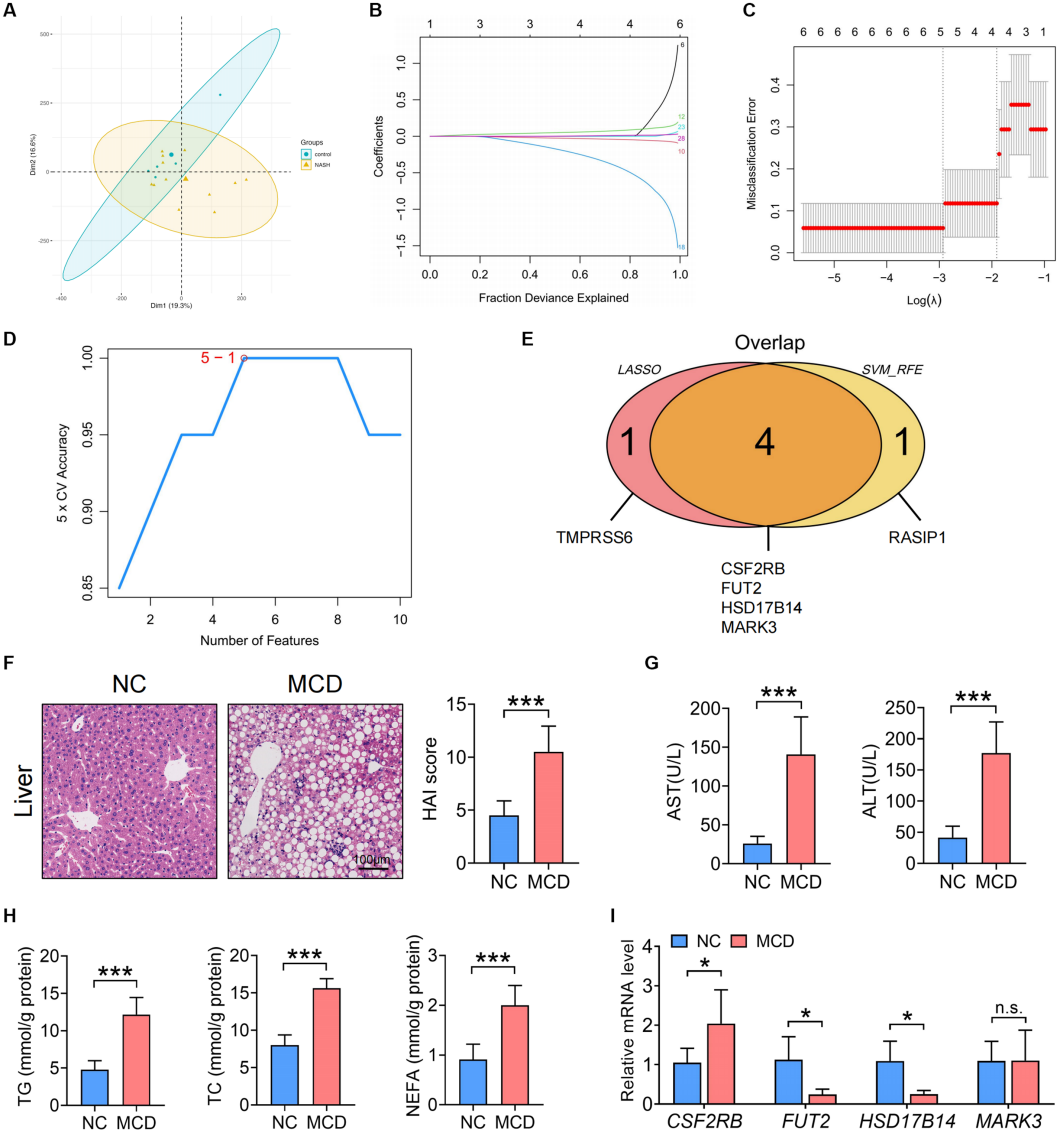
Identification of the key SNP-harboring genes corresponding to the gut microbiota and verification of their expression in the liver and colon of MCD-fed mice with NASH. **(A)** PCA results. **(B)** LASSO coefficient distribution of the differential genes. **(C)** Ten cross-validations of tuning parameter selection in the LASSO model. **(D)** The highest accuracy of the SVM model. **(E)** Wayne graphs for the LASSO and SVM algorithms. **(F)** Representative images of liver hematoxylin and eosin staining and statistical results of the HAI scores of NC or MCD diet-fed mice. Scale =100 μm. **(G)** Serum ALT and AST levels in MCD diet-fed mice. **(H)** Serum TG, TC, and NEFA levels in NC or MCD diet-fed mice. **(I)** qRT-PCR analysis of the key SNP-harbouring genes (*CSF2RB*, *FUT2*, *HSD17B14*, and *MARK3*) in the liver of NC-or MCD diet-fed mice. **(F,I)** *n* = 6 per group. Data are presented as mean ± standard deviation. **(F−H)** Two-tailed unpaired Student’s *t*-test; **(I)** Two-way analysis of variance (ANOVA) followsed by Sidak. MCD, methionine choline-deficient; NASH, nonalcoholic steatohepatitis; PCA, Principal component analysis; SNP, single-nucleotide polymorphism; LASSO, least absolute shrinkage and selection operator; SVM, support vector machine; alanine transaminase, ALT; aspartate transaminase, AST; HAI. Histological Activity Index; TG, triglyceride; TC, total cholesterol; NEFA, non-esterified fatty acid; NC, normal chow.

The IVs in Supplementary Table S1 were converted into 106 genes using the “gwasrapidd” pack-age, the principal component analysis (PCA) diagram shows a significant difference between our control group and the NAFLD disease group (Figure 6A). Further, the corresponding characteristic genes in NAFLD were screened using LASSO regression and the SVM algorithm. After ten cross-validations, LASSO regression identified five NAFLD characteristic genes (Figures 6B,C). However, the top five feature genes with the highest accuracy were screened using the SVM algorithm and intersected with the feature genes screened using LASSO regression (Figure 6D). Four intersection genes were accordingly identified as key genes involved in the regulation of the differential intestinal flora in NAFLD: colony-stimulating factor 2 receptor β (CSF2RB), fucosyltransferase 2 (FUT2), 17-beta-hydroxysteroid dehydrogenase 14 (HSD17B14), and microtubule affinity-regulated kinase 3 (MARK3) (Figure 6E).

To investigate the expression of the aforementioned SNP-harboring genes in the liver and colon of model mice with NASH, C57BL/6 mice were fed an NC or MCD diet for 4 weeks. The MCD diet-fed model mice with NASH exhibited a more notable infiltration of liver inflammatory cells and adipose vacuoles and increased HAI scores than those fed with NC (Figure 6F). Serum ALT, AST, TG, TC, and NEFA levels exhibited a consistent upward trend (Figures 6G,H). Furthermore, the hepatic mRNA expression of *CSF2RB* increased, whereas that of *FUT2* and *HSD17B14* decreased in mice with NASH compared to that in healthy controls (Figure 6I).

## 4 Discussion

NAFLD has emerged as the most common factor contributing to chronic liver disease worldwide, with an increasing global prevalence and an onset at younger ages. Intestinal inflammation, intestinal mucosal barrier destruction, bacterial flora structure disorder, and bacterial metabolite translocation are strongly associated with liver inflammation and fibrosis in NAFLD (Canfora et al., 2019; Tilg et al., 2021). The present study, using bidirectional two-sample MR analysis, revealed a causal relationship between eight intestinal flora and NAFLD and analyzed the target genes regulated by them.

Several previous studies have utilized MR analysis to analyze the causal relationship between gut bacteria and NAFLD. Consistent with these studies, we demonstrated that Lactobacillaceae, *Christensenellaceae R7 group*, and *Intestinibacter* play a protective role in NAFLD, whereas Actinomycetaceae, Oxalobacteraceae, *Actinomycetales* and *Ruminococcaceae UCG005* are harmful bacteria in NAFLD (Li et al., 2023; Zhang R. et al., 2023). These results are consistent with previously reported intestinal microecological changes in NAFLD, with an increase in the abundance of Actinomycetales detected in obese mice fed a high-fat diet (Zhao et al., 2017). The abundances of probiotics, such as Lactobacillaceae (Yu et al., 2021; González-Lozano et al., 2022), *Christensenellaceae R7 group* (Tavella et al., 2021), and *Intestinibacter* (Zhang et al., 2022) were decreased and inversely correlated with the body weight ratio, NAFLD activity score, and visceral and subcutaneous adipose tissue content. In addition, Boursier et al. (2016) have reported that patients with NAFLD and fibrosis have a higher abundance of *Ruminococcaceae* members than those without fibrosis. However, some studies on the abundance and role of *Rumenococcaceae* in NAFLD present divergent findings form our conclusions. For example, the abundance of *Ruminococcaceae* members was low in patients with NAFLD (Tsai et al., 2020). Two studies compared the obesity status of the gut microecology in Latin American NASH and Asian NAFLD cohorts and revealed a significantly lower relative abundance of *Ruminococcaceae* members in non-obese patients with NASH than in non-obese controls (Wang B. et al., 2016; Duarte et al., 2018). Lee et al. (2020) reported that the severity of liver fibrosis in non-obese patients with NAFLD was negatively related to the abundance of *Ruminococcaceae*. In addition, *Faecalibacterium prausnitzii* (*Ruminococcaceae* member) was associated with decreased adipose tissue inflammation in high-fat diet-fed mice (Munukka et al., 2017), and *Ruminococcus faecalis* mitigated fibrosis in diet-induced NAFLD models (Lee et al., 2020). The discrepancies in conclusions may be attributed to the different inclusion criteria for the participants, including the presence of comorbid obesity.

The role of NB1n in NAFLD has not been previously reported, and to the best of our knowledge, this study is the first to identify NB1n as a harmful bacterium in NAFLD. While NB1n has been associated with infections (Lyu et al., 2023; Yan et al., 2023) and urolithiasis (Zhang L. et al., 2023), its role in the development of NAFLD remains unclear. On the other hand, the specific role of Oxalobacteraceae in NAFLD is also completely unclear. Oxalic acid is ingested through the diet or endogenously produced by the liver and metabolized and degraded in the gut by microorganisms, including Oxalobacteraceae members (Ermer et al., 2023). Notably, there is a significant correlation between steatosis severity and oxalate excretion in overweight children and adolescents and higher liver production and urinary oxalate excretion in ob/ob mice (Gianmoena et al., 2021). Furthermore, steatotic hepatocytes are responsible for glyoxalate detoxification (Gianmoena et al., 2021). A recent study revealed that patients with obesity have a significantly higher liver expression of Oxalobacteraceae-specific genes than lean individuals; the genus *Massilia* (Oxalobacteraceae member) was only detected in patients with obesity (Suppli et al., 2021). However, whether Oxalobacteraceae members naturally colonize the liver or translocate from the intestine remains unclear, and their role in oxalic acid synthesis in the liver requires further investigation.

We also demonstrated that the eight target gut bacteria mainly regulate the expression of three key genes in the liver during NAFLD: *CSF2RB*, *HSD17B1*4, and *FUT2.* Compared with the control group, intrahepatic *CSF2RB* mRNA expression increased in the NAFLD group, whereas that of *HSD17B14* and *FUT2* decreased. CSF2RB, also called granulocyte-macrophage colony-stimulating factor/interleukin-3/interleukin-5 receptor common β-subunit, was highly activated in the liver of mice with NASH, aggravating liver tissue inflammation by promoting the production and differentiation of macrophages and granulocytes (Wessendarp et al., 2022). Moreover, the intrahepatic expression of *HSD17B14* (also known as DHRS10, a member of the 17 be-ta-hydroxysteroid dehydrogenase superfamily) was negatively correlated with NASH severity and involved in the regulation of retinol and fatty acid metabolism (Govaere et al., 2020). A large GWAS-based analysis revealed that *FUT2* (rs2519093) mutations are closely associated with low-density lipoprotein levels (Hoffmann et al., 2018). Notably, this gene is highly expressed in the digestive tract (intestine and gallbladder) of both humans and mice and is primarily responsible for encoding alpha-1, 2-focusing transferase (Franks, 2011) and synthesizing H antigen (a receptor for adhesion molecules) (Xing and Guo, 2000). Approximately 20% of White individuals lack the functional *FUT* allele (i.e., the non-secretor), resulting in structural changes in the gut microbiota and disrupting mucosal immunity, which in turn increases the risk of diseases, including inflammatory bowel disease and primary sclerosing cholangitis (McGovern et al., 2010; Folseraas et al., 2012; Jostins et al., 2012). Zhou et al. (2021) have reported that Western diets decrease *FUT*2 expression in the intestinal epithelium of mice and that the loss of Fut2 activity mitigates diet-induced bile acid accumulation, insulin sensitivity, and hepatic steatosis. The specific roles of *CSF2RB*, *HSD17B14*, and *FUT2* in the enterohepatic dialog of NAFLD are unknown, warranting further studies to determine how the eight gut bacteria identified in our study regulate the involvement of these three genes in NAFLD progression.

However, this study has some limitations, necessitating exploration in future research. First, our genome-wide data is limited to European populations, and validating our findings in more diverse populations is critical. Second, several factors, including lifestyle, metabolic factors, age, and sex, that influence the gut microbiota need to be considered (Redondo-Useros et al., 2020; Yuan et al., 2022). Third, the sample size of transcriptome sequencing data needs to be further expanded. Finally, the roles of the eight gut microbes and the related key genes in NAFLD development require further evaluation in animal models.

## 5 Conclusion

Our results suggest a causal relationship between eight gut microbes and NAFLD, highlighting three key SNP-harboring genes (*CSF2RB*, *FUT2*, and *HSD17B14*) for specific microbiome regulation. This study suggests that increasing the abundance of Lactobacillaceae, *Christensenaceae R7 group*, and *Intestinibacter* may alleviate NAFLD and that decreasing that of enterogenous Actinomycetales, NB1n, Actinomycetaceae, Oxalobacteraceae, and *Ruminococcaceae UCG005* may be potential therapeutic targets for NAFLD. In addition, inhibiting the expression of *CSF2RB* or upregulating that of *FUT2* and *HSD17B14* may be another beneficial approach to alleviate NAFLD.

## Data availability statement

The original contributions presented in the study are included in the article/Supplementary material, further inquiries can be directed to the corresponding author.

## Ethics statement

The studies involving humans were approved by the Research Ethics Committee of the University of Tartu and Danish Ethics Committee. The studies were conducted in accordance with the local legislation and institutional requirements. Written informed consent for participation was not required from the participants or the participants’ legal guardians/next of kin in accordance with the national legislation and institutional requirements. The animal study was approved by Institutional Animal Care and Use Committee of Wenzhou Medical University. The study was conducted in accordance with the local legislation and institutional requirements.

## Author contributions

TP: Conceptualization, Writing – review & editing. LS: Data curation, Writing – original draft. YZ: Writing – original draft. FY: Data curation, Writing – original draft. YC: Conceptualization, Funding acquisition, Writing – review & editing.

## Footnotes

^1^ https://www.ncbi.nlm.nih.gov/geo/info/datasets.html
